# Ultrasound elastography in patients with fatty liver disease

**DOI:** 10.1590/0100-3984.2019.0028

**Published:** 2020

**Authors:** Luiza de Campos Moreira da Silva, Julia Teixeira de Oliveira, Sandra Tochetto, Claudia Pinto Marques Souza de Oliveira, Rosa Sigrist, Maria Cristina Chammas

**Affiliations:** 1 Faculdade de Medicina da Universidade de São Paulo (FMUSP), São Paulo, SP, Brazil.

**Keywords:** Elasticity imaging techniques/methods, Ultrasonography/methods, Liver/diagnostic imaging, Fatty liver/diagnosis

## Abstract

Hepatic steatosis, or fatty liver disease, occurs due to the accumulation of lipids in hepatocytes. When it becomes chronic, lobular inflammation develops and the disease can evolve to hepatic fibrosis, liver cirrhosis, or hepatocellular carcinoma. Early diagnosis is desirable because patients diagnosed in the early stage of the disease respond better to treatment. In the early stages of fatty liver disease, the physical examination is often unremarkable. Fatty liver disease and hepatic fibrosis can be diagnosed and monitored through laboratory tests, imaging, and biopsy. Among the imaging methods, ultrasound stands out as an effective means of diagnosing and following patients with liver disease. Ultrasound used in conjunction with elastography (ultrasound elastography) has recently shown great utility in the follow-up of such patients. Ultrasound elastography studies the degree of deformation (stiffness) of an organ or lesion, so that when there is hardening, fibrosis, or cirrhosis of the liver, those alterations are well demonstrated. In this review article, we discuss the application of the different types of ultrasound elastography for liver studies: transient elastography, point shear wave elastography, and two-dimensional shear wave elastography. Although magnetic resonance elastography may also be used in the analysis of liver fibrosis, it will not be addressed in this article.

## INTRODUCTION

Hepatic steatosis, or fatty liver disease, occurs as a result of the deposition and accumulation (to over 5% of the weight of the liver) of microvesicular or macrovesicular lipids (typically triglycerides) in hepatocytes. The main risk factors for the condition are diseases that lead to metabolic disorders, such as obesity, type 2 diabetes mellitus, and hyperlipidemia, and other comorbidities that damage hepatocytes, such as hepatitis C, as well as the use of medications and alcohol consumption (Table 1). 

**Table 1:** Causes of NAFLD.

Insulin resistance	Obesity, type 2 diabetes mellitus, dyslipidemia
Metals	Antimony, barium, borates, phosphorus, chromates, carbon disulphide, thallium compounds, and uranium compounds
Cytotoxic drugs	L-asparaginase, azacytidine, azauridine, methotrexate, glucocorticoids, antiretroviral, amiodarone, tamoxifen, estrogens, war-farin, bromoethane, hydrazine, 5-fluorouracil, tetracycline, bleomycin, azaserine
Inborn errors of metabolism	Wilson's disease, familial steatohepatitis, galactosemia, hereditary fructose intolerance, homocystinuria, systemic carnitine deficiency, tyrosinemia, Refsum disease, Shwachman syndrome, Weber-Christian syndrome
Other	Hepatitis C, HIV, inflammatory bowel disease, cachexia, total parenteral nutrition, diverticulitis, autoimmune hepatitis

Nonalcoholic fatty liver disease (NAFLD), unlike other liver diseases, is not related to excessive alcohol consumption, rather being related to metabolic syndrome, insulin resistance being one of the major predisposing factors^(1)^. Increases in the incidence of obesity, type 2 diabetes mellitus, and metabolic syndrome have contributed to the increased incidence of NAFLD, so that its prevalence now ranges from 15% to 24%, depending on the country^(2,3)^. It affects an estimated 57.5-74.0% of obese individuals^(2,4,5)^, 22.5% of children^(6)^, and 52.8% of obese children^(7)^. It is also estimated that 50% of individuals with type 2 diabetes mellitus have some degree of NAFLD^(2,7)^. Patients with the chronic form of NAFLD develop lobular inflammation, and the disease can evolve to hepatocyte ballooning and fibrosis, conditions collectively known as nonalcoholic steatohepatitis (NASH). As the fibrosis advances, NAFLD evolves to cirrhosis and, subsequently, to hepatocellular carcinoma^(8)^.The accurate diagnosis of fibrosis and hepatic inflammatory activity is extremely important for determining the stage and prognosis of the disease, as well as for planning the treatment^(9)^.

Although serology and the identification of biological markers have little value in determining the degree of fibrosis, their significance increases when they are combined with other tests. Histopathological analysis of a liver biopsy specimen continues to be the gold standard for the diagnosis and staging of fibrosis. However, liver biopsy has relevant limitations and complications, which has led to the development of noninvasive methods, such as imaging examinations, to estimate the degree of liver fibrosis^(10)^.

This article aims to review the various diagnostic ultrasound methods used in determining the degree of fibrosis and the extent of hepatic involvement in patients with fatty liver disease.

## DIAGNOSTIC METHODS

Accurate, early diagnosis of liver disorders is desirable because it provides a better therapeutic response in patients in the early stage of fatty liver disease. To that end, there are a number of methods for analyzing the liver^(11)^.

The evaluation and diagnosis of steatosis and liver fibrosis can be performed by ancillary examinations: laboratory tests, imaging, and biopsy. Clinical examination does not allow early diagnosis of liver abnormalities, because most patients in the early stage of fatty liver disease are asymptomatic, showing no alterations on physical examination. Imaging examinations include conventional ultrasound, as well as ultrasound-based methods known as transient elastography (TE), point shear wave elastography (pSWE), and two-dimensional shear wave elastography (2D-SWE). Another such examination is strain elastography, a form of quasi-static ultrasound-based imaging. However, strain elastography is more effective in superficial tissues and is therefore not used for imaging of the liver. Magnetic resonance elastography (MRE) can also be used for the analysis of liver fibrosis. In this context, liver biopsy is still considered the gold standard for the diagnosis of fatty liver disease^(10)^.

### Biomarkers of steatosis and liver fibrosis in NAFLD

In most cases, NAFLD is suspected or discovered by chance in routine evaluations, usually simple abdominal ultrasound or laboratory tests focused on liver enzymes. The first-line examination is abdominal ultrasound, because it is inexpensive and widely available. It should be noted, however, that ultrasound has low sensitivity for the detection of steatosis when it affects < 20% of the liver or in individuals with a body mass index (BMI) > 40 kg/m2 (class III obesity). Magnetic resonance spectroscopy, the ideal imaging examination for the detection of fatty liver disease, is costly, therefore being used only at specialized centers and for research purposes. Another noninvasive approach to NAFLD is the use of clinical scores for steatosis, and three such scores have gained relevance because they have been externally validated, although they serve only to establish presence, rather than assessing severity^(12,13)^ : the fatty liver index, the SteatoTest, and the NAFLD liver fat score. For fibrosis, a major determinant of the prognosis of NAFLD, biomarkers are less accurate in detecting the intermediate stages than in detecting cirrhosis.

In 65-90% of healthy subjects, the aspartate aminotransferase/alanine aminotransferase (AST/ALT) ratio is less than 1. In patients with steatosis, the AST/ALT ratio begins to increase, mainly due to increased AST levels; that trend inverts in those who develop marked fibrosis, those in whom the disease progresses, and those who evolve to cirrhosis. In some cases of advanced fibrosis (< 50% of cases), alkaline phosphatase and gamma glutamyltransferase levels are two to three times higher than the reference value. In addition to those markers, other variables, such as age, glycemic index, BMI, platelet count, and albumin level, are used for the analysis of fibrosis^(14)^.

The investigation of steatosis requires tests to identify markers of various states^(15)^ : oxidative stress (low plasma levels of vitamin E, inactivation of glutathione peroxidase, and detection of exhaled nitric oxide or volatile organic compounds on a breath test); inflammation (elevated serum levels of cytokines such as tumor necrosis factor alpha and adiponectin); and apoptosis. In the NASH test, we look for increases in alpha-2 macroglobulin, apolipoprotein, total bilirubin, and gamma glutamyltransferase^(16)^.

In addition to the biomarkers described, there are several scales for the diagnosis of fibrosis: the hypertension, ALT, and insulin resistance (HAIR) score; and the BMI, age, ALT, and triglyceride (BAAT) score. The HAIR score is used in order to determine whether or not a patient has NAFLD on the basis of the parameters arterial hypertension, elevated ALT, and insulin resistance. The presence of two or more factors indicates NASH^(17)^. The BAAT score classifies patients as potentially having liver fibrosis or liver cirrhosis if they meet at least one of the following criteria^(16)^ : BMI > 28 kg/m2; age ≥ 50 years; ALT ≥ 2 times above normal; and triglycerides ≥ 1.7 mmol/L. The HAIR and BAAT scores both serve to identify the predictors of liver fibrosis and NASH severity. However, the instruments that currently have the best external validation are the NAFLD fibrosis score, the FIB-4 index^(18)^, and the FibroTest, the last having previously been used in order to detect advanced fibrosis in patients with chronic hepatitis B or C. Those tests are highly accurate in distinguishing advanced fibrosis-that categorized as stage 3 (F3) in the METAVIR scoring system-from the milder forms.

Another noninvasive tool for the assessment of fibrosis is TE (FibroScan; Echosens, Paris, France), which is already widely used in chronic viral hepatitis. Like other tests, TE is better at differentiating between advanced and mild fibrosis. The controlled attenuation parameter (CAP) can be measured with elastography and has been shown to detect steatosis with good accuracy, although it is less reliable for determining the degree of involvement^(19)^.

There is still no consensus on the use of markers of steatosis and liver fibrosis for the initial and continued evaluation of NAFLD, especially in terms of their effectiveness in avoiding liver biopsy. Many authors have suggested combining several of these diagnostic methods.

### Liver biopsy

To confirm the diagnosis of NASH, a biopsy is still required. The decision to propose a biopsy should be discussed with and individualized for each patient. A number of clinical findings associated with NASH, as well as advanced fibrosis in patients with NAFLD, can facilitate the indication of liver biopsy, namely being over 45 years of age, being obese or having diabetes, and having an AST/ALT ratio > 1. The indications for liver biopsy are as follows: altered liver enzymes or fibrosis scores in the context of steatosis (usually seen on ultrasound); and to determine the predominant type of liver involvement in patients with concomitant diseases.

Histopathological analysis is still the ideal procedure for staging the disease. The typical findings of NASH include steatosis, which is usually macrovesicular and graded as one of three levels; hepatocyte ballooning; mixed lobular inflammatory infiltrate; and fibrosis, which is initially perisinusoidal, predominantly in zone 3 (centrilobular zone), although it can also be seen in the periportal zone. However, fibrosis is not a necessary condition for diagnostic confirmation. In addition to these findings, there can be periportal inflammation, glycogen-type vacuolization, megamitochondria, and fewer Mallory-Denk bodies than in the alcoholic form of the disease, which is in many respects identical, although it features more pronounced lobular inflammation and other characteristics^(19)^.

Although biopsy is the gold-standard method for the diagnosis of NAFLD, there is controversy regarding its validity. One issue is the representativeness of the material, given that the typical liver fragment analyzed has a volume of only approximately 1/50,000 of the total organ volume. Therefore, the extent of tissue damage can be underestimated, because the process of parenchymal fibrogenesis is dynamic and has a heterogeneous distribution. In addition, there is some interobserver variation in the interpretation of slides and there can be disagreement among pathologists, especially among those with less experience. Several studies have shown that interobserver agreement is almost perfect among more experienced pathologists. Therefore, the kappa statistic for interobserver agreement can range from 0.4 to 0.9^(8)^. Because liver biopsy is an invasive procedure, it carries a risk of serious complications, which occur in approximately 1% of cases^(20)^. Therefore, in the context of the follow-up of individuals with NAFLD, it is not the most appropriate examination, although it best reflects the degree of tissue damage.

### Ultrasound

Ultrasound is the simplest diagnostic method, providing the best diagnostic results when steatosis affects more than 30% of the liver^(21)^. It is noninvasive, easily accessible, and cost effective, revealing aspects suggestive of steatosis in more than 16% of healthy nonobese individuals and in approximately 95% of obese people who consume alcohol. However, it has limited sensitivity to detect steatosis that affects less than 20% of the liver and to detect steatosis in obese patients with a BMI > 40 kg/m2. Studies have shown good agreement between the ultrasound classification and the histopathological stage, as well as showing that, for the detection of steatosis, ultrasound has a sensitivity of 89% and a specificity of 93%^(22)^. However, it is not possible to differentiate between NASH and NAFLD steatosis by ultrasound, histopathological analysis being required in order to make that distinction^(7)^.

**Figure 1:**
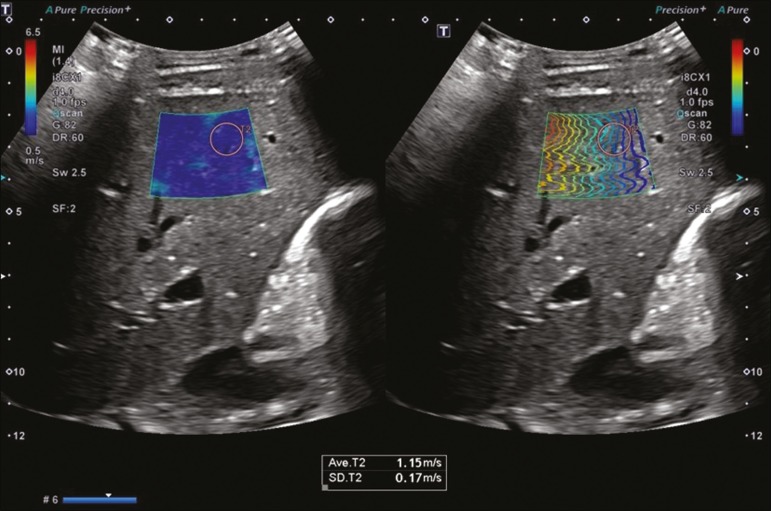
Ultrasound examination of a normal liver. A 2D-SWE study showing normal elasticity of the liver parenchyma (1.15 m/s), consistent with a METAVIR stage of F0. The elastography map is homogeneous and predominantly blue, a color range that represents less stiffness, and the propagation mode (on the right) indicates normality.

In the quantitative ultrasound classification system devised by Saadeh et al.^(22)^, steatosis can be categorized as follows (Figures 1 through 5): grade 1 (mild)-mild, diffuse increase in hepatic echogenicity, the hepatic vessels and diaphragm having a normal aspect; grade 2 (moderate)-moderate, diffuse increase in hepatic echogenicity, the hepatic vessels and diaphragm having a blurred aspect; grade 3 (marked)-marked increase in hepatic echogenicity, the hepatic vessels, diaphragm, and posterior liver not being visible^(22,23)^.

### Elastography

Elastography is a method that quantifies liver fibrosis by measuring the propagation velocity of ultrasound waves that pass through the liver; as fibrosis progresses, the liver tissue stiffens and the waves propagate faster. On the basis of the wave propagation velocity, it is possible to determine the degree of stiffness and therefore the stage of liver fibrosis. There are several types of ultrasound elastography, the main ones used for the study of the liver being TE, 2D-SWE, and pSWE.

#### Examination technique

For all elastography examinations, regardless of the method employed, the patient should be in the supine or left lateral position, with the right arm raised above the head to broaden the intercostal acoustic window, and the transducer should be positioned over the intercostal space^(24)^. The location selected for the measurement should take into account the depth and choice of the most appropriate acoustic window to achieve the most reliable measurement of liver stiffness, provided by multiple measurements taken at the same location. To optimize the results, the measurement is taken during a brief breath hold (of a few seconds), because deep breathing and the Valsalva maneuver both change the hepatic venous pressure and can thus alter the assessment of stiffness^(8)^.

Previous studies of elastography suggest that 10 measurements should be obtained and that the mean of those measurements should be recorded. Measurements deemed acceptable should account for at least 60% of the measurements. An acceptable measurement is defined as one that produces a numerical result other than “x.xx” or “0.00”^(8)^. The interquartile/median ratio is the statistical measure of dispersion, being equal to the difference between the largest and the smallest quartile, and reflects the variability of valid measurements^(8,25)^ : an interquartile/median ratio < 0.30 indicates that the data are valid.

## TE

The FibroScan device is used in performing TE. It was the first device developed to measure liver elasticity as an alternative to biopsy. It is dedicated exclusively to the analysis of liver fibrosis, having no other diagnostic applications because it is not a conventional ultrasound device. It employs an ultrasound transducer operating at 5 MHz, which is built onto the shaft of a piston that acts as a vibrator. At the push of a button, low frequency (50-Hz) transient vibrations are transmitted and the elastic shear waves generated propagate through the underlying tissues. It is used in order to assess tissue elasticity, which allows the degree of fibrosis to be estimated, in patients with chronic liver disease^(25)^.

**Figure 5:**
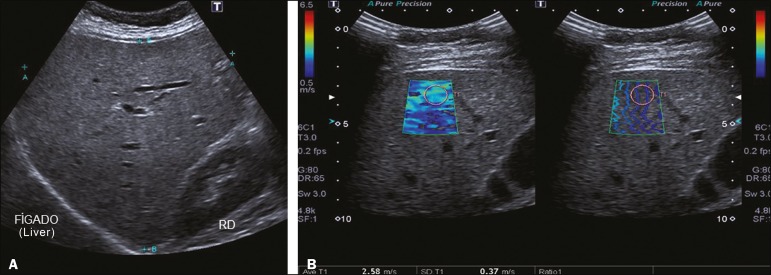
**A:** Ultrasound examination of a liver with mild steatosis and signs of chronic liver disease, as characterized by blunt edges, irregular contours, and heterogeneous parenchyma. **B:** A 2D-SWE study showing greater stiffness of the liver parenchyma (2.58 m/s), consistent with a Metavir stage of F4. On the elastography map, tones of green predominate, and there is greater line separation in the propagation mode.

Ultrasound acquisitions are used in order to monitor the propagation of the shear wave and to measure its velocity. In a TE examination, liver stiffness is measured in a cylindrical volume of approximately 10 × 40 mm at a depth of 25-65 mm below the skin surface, which is at least 100 times larger than a biopsy specimen and therefore much more representative of the liver parenchyma^(9)^. Measurements should be performed in an area of parenchyma without vessels or bone; those acquired inappropriately or with excess transducer pressure on the skin are automatically discarded.

The intensity of the ultrasound wave is directly related to the elasticity, decreasing exponentially as it propagates in the medium; that is, the stiffer the tissue is, the faster the vibrations will propagate. Therefore, the higher the result is (in kPa), the greater is the degree of parenchymal fibrosis in the liver^(25)^.

The CAP is an operator-independent tool associated with the TE device and is used in order to quantify hepatic steatosis. The measurements obtained by CAP (in dB/m) follow the same acquisition pattern as those obtained by FibroScan. A study of overweight and obese patients with chronic liver disease reported that CAP determination showed sensitivity and specificity of 76% and 79%, respectively, for the detection of steatosis involving less than 10% of hepatocytes (214-289 dB/m), 85% and 79%, respectively, for the detection of that involving 11-33% of hepatocytes (233-311 dB/m), and 83% and 79%, respectively, for the detection of that involving 67-100% of hepatocytes (266-318 dB/m). Although there have been few studies evaluating the CAP method, it has proven to be a good way to evaluate and monitor patients with NAFLD, because it is easily performed and able to analyze a portion of the liver 100 times larger than the typical liver biopsy specimen, with the additional advantages of being operator independent and providing immediate results^(23)^. Studies show that FibroScan has good reproducibility, although the level of agreement decreases in patients with less fibrosis, less steatosis, or a high BMI^(7)^.

### pSWE

The pSWE technique consists in measuring the velocity of shear wave propagation in the liver parenchyma as a way of assessing the degree of stiffness of the liver^(26)^. An acoustic radiation force impulse (ARFI) is applied in a region of interest chosen by the operator. Unlike TE, pSWE does not require vibratory stimulation, making the measurement more accurate, with less interobserver variation and greater reproducibility^(25,27)^ . The area analyzed is a 10 × 5 mm rectangle that can be freely moved in the two-dimensional (B-mode) image to a maximum depth of 80 mm below the skin surface^(25)^, which allows a more appropriate measurement in obese patients and in patients with ascites. Measurements are preferably performed in segments 5 and 8 of the right lobe of the liver, in the intercostal spaces. Examination of the left lobe by pSWE is useful in obese patients, in whom there can be technical difficulties in obtaining measurements in the right lobe.

The pSWE technique provides a numerical indication of the degree of liver stiffness by emitting acoustic pulses that produce shear waves, whose velocity is proportional to the degree of stiffness of the examined organ (higher velocity equals greater stiffness). As steatosis progresses to fibrosis, the stiffness of the organ increases, resulting in higher shear wave propagation velocity. In addition, because pSWE is a technique coupled with conventional ultrasound equipment, it is possible to evaluate the morphology of the liver (in B mode), to perform a Doppler study of the liver, and to avoid areas such as vessels and other structures that could compromise the procedure and distinguish between stiffened and preserved areas when using a gray scale or color scale, thus allowing a more comprehensive evaluation of the liver in comparison with the TE technique^(26)^.

For staging fibrosis, the histological scoring system of choice is the METAVIR system (Table 2), in which the results obtained by pSWE are compared with those obtained by liver biopsy. Studies have shown that pSWE has good sensitivity and specificity in comparison with biopsy^(24)^. A meta-analysis of a collective sample of 518 patients showed a correlation between pSWE and liver biopsy, the former showing an accuracy of 87% for F2 fibrosis, 91% for F3 fibrosis, and 93% for F4 fibrosis^(20)^.

**Table 2:** Morphological criteria for staging fibrosis with the METAVIR histological scoring system^(28)^.

Histopathological finding	Stage
No fibrosis	F0
Portal fibrous expansion without septa	F1
Fibrous expansion with a few septa	F2
Fibrous expansion with many septa, without cirrhosis	F3
Cirrhosis	F4

The high cost of the equipment used in pSWE restricts the application of the technique beyond referral centers. However, for some ultrasound systems, it is possible to update the software or hardware to implement the elastography tool without replacing the equipment, which could reduce the budgetary impact of the eventual incorporation of this technique at smaller facilities.

## 2D-SWE

In the 2D-SWE technique, multiple ARFI measurements are made over a large field of view, which can be achieved with a single real-time image. The mean, maximum, minimum, and standard deviation of the shear wave velocity are analyzed in one region of interest. Because 2D-SWE is performed in real time, it evaluates various regions of the liver and allows the production of elastography measurements to be visualized on a color display as they accumulate^(20,25)^. The ARFI focus is dragged below the acoustic axis faster than the shear wave velocity, in order to generate almost simultaneous tissue shifts (in the tens of micrometers) at all positions along the acoustic axis, producing a shear wave in the form of a shallow-angle cone that shifts from the pressure line, propagating less and thus decaying less rapidly with distance than it would from a single pushing focus.

An ultrafast scanner achieves an ultrasound rate of up to 20 kHz, transmitting a flat wave and focusing only on receiving, so that each ultrasound echo image is created with a single push pulse. The differences between the arrival times at different positions are then analyzed to create a portion of the final shear wave velocity image. The process is repeated over a number of different push lines to create a final quantitative image of elasticity in a box, which is presented as a color overlay on the B-mode image (in m/s) or converted to Young’s modulus (in kPa, as for TE). Therefore, the software uses three related frame rates^(27)^ : the standard B-mode echo image; an echo image used in order to track displacement; and the shear wave elastogram.

In patients with chronic liver disease, the 2D-SWE technique has shown greater accuracy in detecting the early and intermediate stages of fibrosis than has the TE technique. Although 2D-SWE is only minimally operator dependent, care must be taken to minimize transducer pressure to avoid the overestimation of stiffness during surface tissue imaging. Factors that affect data quality, producing speed errors or signal loss, include the following^(24)^ : the strength and speed of the shear wave; variations in the attenuation, absorption and reflection of the pushing beam; ultrasound scatterer spacing; tissue continuity; and scattering, reflection, or refraction of the shear waves.

It is noteworthy that the various ultrasound elastography systems marketed by different manufacturers have different cutoff values (in m/s or kPa) for each liver fibrosis stage. Table 3 shows how the different techniques relate to the METAVIR classification^(29)^. Figures 1 through 5 provide examples of the results obtained with 2D-SWE.

**Table 3:** Cut-off values for different stages of liver fibrosis determined by various ultrasound elastography techniques and equipment marketed by various manufacturers^(29)^.

Ultrasound elastography techniques	≥ F2	≥ F3	F4
pSWE (VTQ/ARFI) - Siemens	1.34 m/s	1.55 m/s	1.80 m/s
pSWE (ElastPQ) - Philips*	1.37 m/s	2.00 m/s	2.64 m/s
2D-SWE - General Electric*	1.66 m/s	1.77 m/s	1.99 m/s
2D-SWE (SSI) - Aixplorer	1.50 m/s	1.70 m/s	1.90 m/s
2D-SWE (ASQ) - Toshiba*	1.76 m/s	2.21 m/s	2.86 m/s
TE-FibroScan - Echosens	1.67 m/s	1.70 m/s	2.10 m/s

* Cut-off values provided by the manufacturer.

## WHAT IS CONSOLIDATED IN THE LITERATURE

The literature indicates that ultrasound elastography can be used in order to distinguish between patients without fibrosis and those with mild fibrosis, as well as between those with mild fibrosis and those with severe fibrosis or cirrhosis, without the need for an invasive procedure unless there is a concomitant disease factor, such as a risk of acute hepatitis, that would not be definitively diagnosed with a noninvasive method^(8)^. In addition, the efficacy of ultrasound elastography depends on physician knowledge and the technique employed in the examination, which is made more difficult by the complexity of liver disease and the variety of techniques available.

The Brazilian Hepatology Society issued a consensus that guides practice in the diagnosis of NAFLD in Brazil^(30)^. The consensus emphasizes the importance of investigating metabolic factors and diseases that can lead to steatosis, recommending a thorough clinical evaluation and the ordering of laboratory tests (of liver function and enzymes, as well as serology). According to the consensus, diagnostic imaging methods such as ultrasound, computed tomography, and magnetic resonance imaging are classified as ancillary to biopsy, because they are important for excluding differential diagnoses but are not able to distinguish between steatosis and steatohepatitis. Fortunately, TE and other ultrasound elastography methods are described as contributing to the diagnosis of liver fibrosis in NAFLD patients. Biopsy is strongly indicated only in the following cases: suspected steatohepatitis with various differential diagnoses (other chronic liver diseases); NAFLD at high risk of steatohepatitis or advanced fibrosis, suggested by serological markers or elastography findings; elevated liver enzymes (AST or ALT) for more than three months; and metabolic syndrome that is not controlled after six months of nonpharmacological treatment and lifestyle modification.

The complexity of the diagnosis of liver fibrosis and NAFLD is viewed with enthusiasm by the research community, because it creates opportunities for lines of research in this area. As a result, ultrasound elastography is moving toward considerable improvements in the current techniques. Major improvements are expected in terms of image quality, ease of use, quantification, and range of measurable tissue characteristics. It may one day completely replace liver biopsy in the diagnosis of steatosis, saving many individuals from undergoing this invasive procedure^(20,27)^.

## Figures and Tables

**Figure 2:**
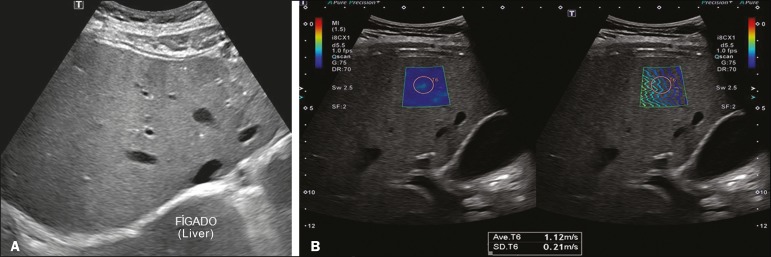
**A:** Ultrasound examination of a liver with mild steatosis, as characterized by the slightly increased echogenicity of the liver parenchyma. The hepatic vessels and diaphragm are clearly visible. **B:** A 2D-SWE study showing normal elasticity of the liver parenchyma (1.12 m/s), consistent with a METAVIR stage of F0. The elastography map is homogeneous and predominantly blue, indicating less stiffness. The propagation mode (on the right) indicates normality.

**Figure 3:**
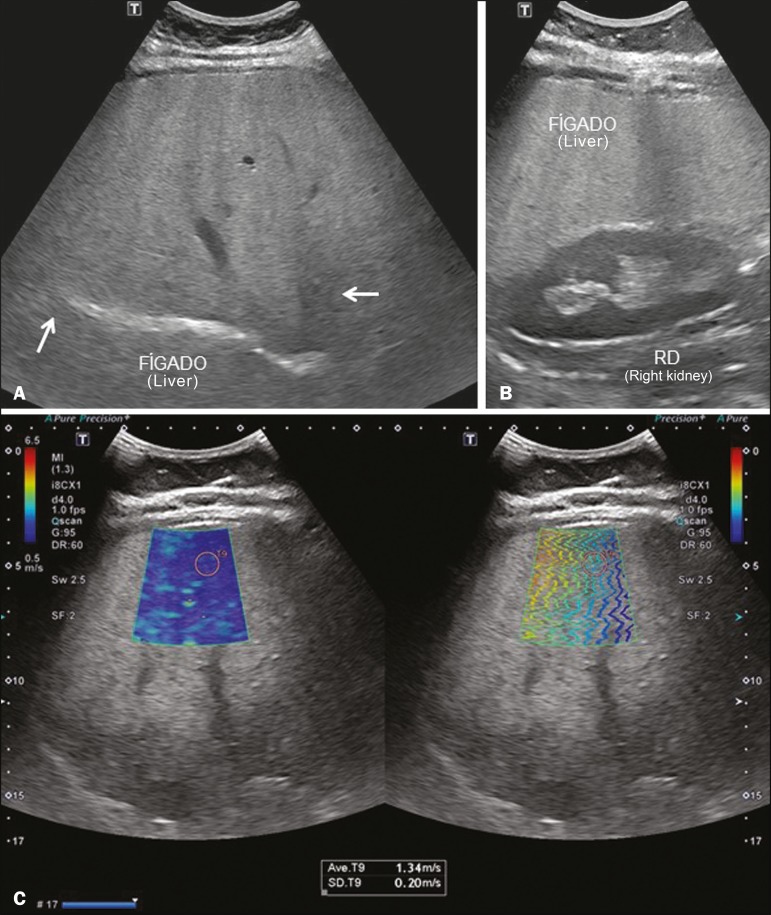
**A:** Ultrasound examination of a liver with moderate steatosis, as characterized by increased echogenicity of the liver parenchyma and posterior attenuation of the ultrasound beam. The attenuation of the ultrasound beam decreases the definition of the hepatic vessels and the diaphragm, although they are still identifiable (arrow). The echogenicity of the liver is higher than is that of the right kidney (**B**). **C:** A 2D-SWE study showing greater parenchymal stiffness, albeit still within normal limits (1.34 m/s), consistent with a METAVIR stage of F0/F1. Although the elastography map is a bit less homogeneous, it is still predominantly blue, indicating less stiffness, despite a larger number of green areas.

**Figure 4:**
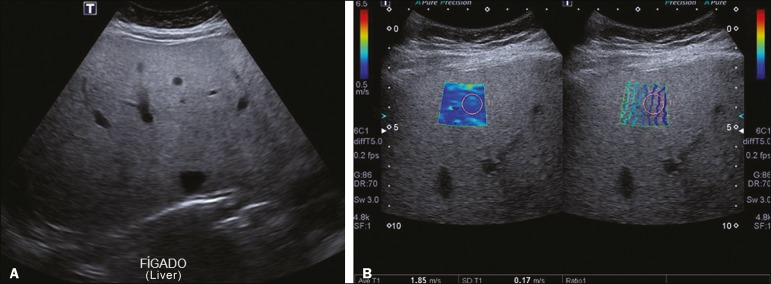
**A:** Ultrasound examination of a liver with marked steatosis, as characterized by increased echogenicity of the liver parenchyma and posterior attenuation of the ultrasound beam. Attenuation of the ultrasound beam obscures the view of the diaphragm. **B:** A 2D-SWE study showing greater stiffness of the liver parenchyma (1.85 m/s), consistent with a Metavir stage of F3. The blue and green tones are in equal proportions on the elastography map and the lines are further apart in the propagation mode.
